# Blighted ovum and tubal pregnancy: a rare form of heterotopic pregnancy: case report

**DOI:** 10.1186/s13104-018-3345-2

**Published:** 2018-04-12

**Authors:** Desmond Aroke, Larry Tangie Ngek, Maxime Tindong, Esua Fomanka, Colette Achu, Arnold Agwe Tanah, Benjamin Momo Kadia

**Affiliations:** 1 Mbengwi District Hospital, Mbengwi, Cameroon; 2 Health and Human Development (2HD) Research Network, Douala, Cameroon; 3 Etoug-Ebe Baptist Hospital, Yaounde, Cameroon; 4 Universite Libre de Bruxelles, Brussels, Belgium; 5 Faculty of Health Sciences, University of Buea, Buea, Cameroon; 6 Foumbort District Hospital, Foumbort, Cameroon; 7 Grace Community Health and Development Association (GRACHADA), Kumba, Cameroon

**Keywords:** Blighted ovum, Ectopic pregnancy, Case report

## Abstract

**Background:**

Heterotopic pregnancies are rare in spontaneous conceptions. Nonetheless, when it does occur, the intrauterine pregnancy is usually viable. We herein present a true rarity of the coexistence of a blighted ovum and an ectopic pregnancy.

**Case presentation:**

A 25 year old G2P1001 married seamstress of African ethnicity at 8 weeks of amenorrhoea presented to our health facility with a 4 day history of lower abdominal pains and vaginal bleeding for which physical examination revealed a closed cervix. Trans-abdominal ultrasound scan confirmed a diagnosis of a blighted ovum and an ectopic pregnancy. Patient was managed with surgical therapy. Evolution thereafter was uneventful.

**Conclusion:**

The case presented confirms that HP can occur in the absence of predisposing factors, and that the detection of a blighted ovum should not preclude the possibility of a simultaneous ectopic pregnancy. A high index of suspicion could lead to early diagnosis, prompt management and a favourable prognosis even in a low-income setting.

## Background

Heterotopic pregnancy (HP) is often used to describe the coexistence of an intrauterine and an ectopic pregnancy [1]. It is a seldom but yet fatal condition whose diagnosis can easily be missed. Heterotopic pregnancies are thought to be caused by multiple ovulations; the incidence is thus expected to be higher amongst women with assisted reproductive techniques [2–4]. The incidence of HP in the general population is estimated to be 1 in 30,000. However, incidence as high as 1% have been reported in patients with assisted reproduction; induction with clomiphene citrate, in vitro fertilization etc. [1, 3]. The ectopic component of HP could be a living or dead foetus found either in the cervix, fallopian tubes, ovaries or even intra-abdominal. Likewise, the intrauterine pregnancy could either be dead or alive in the uterine cavity [5–7]. However, not much has been reported on a blighted ovum as the intrauterine component of an HP.

We report the case of a patient with the coexistence of an intrauterine and extrauterine pregnancy, with the intrauterine component being a blighted ovum.

## Case presentation

A 25 year old G2P1001 sub-Saharan African married seamstress at 8 weeks amenorrhea, presented to a primary level hospital in Cameroon with 4 days history of lower abdominal pains and vaginal bleeding. She had no known chronic illness and denied having any past history of pelvic inflammatory disease or assisted reproduction.

She reported being well till 4 days prior to presentation when she started experiencing abdominal pain; the pain was mainly in her lower abdomen, dull in nature, non-radiating, mild in intensity and was initially intermittent then became constant. It was associated with mild per vagina loss of bright red blood. She had no other symptoms. This prompted her to consult at a drug store, where she was prescribed phloroglucinol tablets 80 mg/day in 2 divided doses which she took for 3 days but had no regression of the symptoms. The persistent lower abdominal pain and mild vagina bleeding prompted a second consultation at our health facility. Upon presentation she was haemodynamically stable and vaginal examination was relevant for a closed cervix. A presumptive diagnosis of threatened abortion with possible aetiology of a urinary tract infection was made. Urinalysis showed leucocyturia while obstetric echography revealed an empty gestational sac measuring 29.9 mm. Diagnoses of blighted ovum and urinary tract infection were made. She was given a short course of antibiotics and programmed for manual vacuum aspiration. Vacuum aspiration was done and the patient served a single intramuscular dose of 10 units of oxytocin. The bleeding and lower abdominal pain stopped and she was discharged the next day.

Two weeks following discharge lower abdominal pain reoccurred. This time, the abdominal pain was constant and localized to the left lower quadrant. It was associated with intermittent episodes of bright red vaginal bleeding. These symptoms persisted for 3 days and prompted another consultation.

On arrival she had a good general state. Her blood pressure was 102/64 mmHg, heart rate 88 beats/min, respiratory rate of 20 breaths/min, temperature 37.2 °C, O_2_ saturation at 97% and weight 58 kg. Her conjunctivae were pink and no scleral icterus, heart sounds were normal and lung fields clear. Her abdomen was flat, moved with respiration and there were no scars. There was no tenderness on superficial palpation but left iliac fossa tenderness on deep palpation. The liver and spleen were not palpable. A speculum examination was unremarkable but for bright red blood oozing out of the cervical os. Vaginal examination revealed a firm closed posterior cervix with left adnexal tenderness on bimanual palpation. The limbs were without particularity.

Based on these findings a tentative diagnosis of an associated ectopic pregnancy was made. A repeat pelvic ultrasound revealed: a homogenous uterus; left adnexal mass measuring 58 mls; pouch of Douglass collection. A repeat positive pregnancy test and sonographic findings confirmed a diagnosis of ectopic pregnancy.

The patient was immediately planned for an emergency exploratory laparotomy indicated for a possible ectopic pregnancy. Preoperative work up included: normal haemoglobin of 11.7 g/dl; normal white cell count of 8100/µl; normal platelet count of 320,000/µl, normal kidney function test (serum creatinine of 0.64 mg/dl and urea of 12.7 mg/dl), glycaemia of 85.9 mg/dl and normal serum electrolytes of: (sodium 134 mmol/l, potassium of 4.17 mmol/l and chloride of 103 mmol/l).

**Fig. 1:**
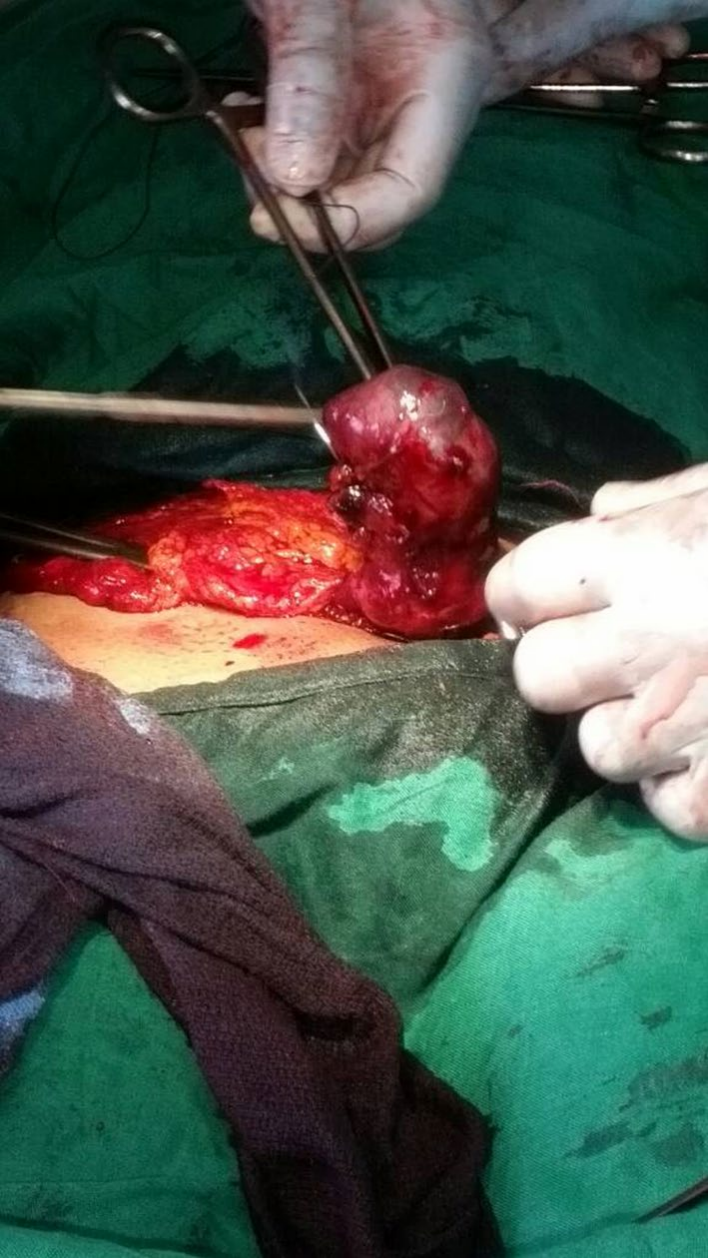
Picture depicting intraoperative left unruptured tubal pregnancy

An emergency laparotomy was performed under general anaesthesia. Intra-operative findings revealed unruptured left tubal pregnancy (Fig. 1) in the ampulla. A left salpingectomy was performed with excision of the unruptured fetus. The right tube was inspected and found to be normal. The abdomen was then closed layer by layer. The patient was monitored in the recovery room for 1 h during which she was haemodynamically stable. She was sent to the ward where close monitoring continued.

Postoperatively, the patient was maintained on nil per os, intravenous infusions of dextrose–saline, intravenous prophylactic antibiotics and analgaesics. Early ambulation was encouraged upon recovery from general anaesthesia. Routine postoperative care in the ward continued and was uneventful. She was discharged on post operation day 7. Evolution thereafter was favourable.

## Discussion and conclusions

HP is the simultaneous presence of intrauterine and extrauterine pregnancies. The existence of HP is unusual in natural conception cycles with a reported incidence of 0.08%. However this incidence increases to up to 1% in assisted reproductive techniques and 5% of pregnancies following in vitro fertilization. HP is thus rarely considered as a differential of first trimester bleeding following natural conception as was in our case, and thus likely to be missed [1, 8]. Furthermore, research shows that the main risk factors associated with ectopic pregnancy are tubal surgery and pelvic inflammatory disease [1, 2], which were nonexistent in our patient. The suspicion of HP in this patient was thus very unlikely.

In HP, the ectopic pregnancy could be cervical, tubal, ovarian, on broad ligament or intraabdominal [9, 10]. Of these, 95–97% are tubal with the most common site being the ampulla (80%), followed by the isthmic segment of the tube (10%), then fimbriae (5%) and the corneal and interstitial segments (2–4%) [11]. As described in literature the ectopic component of this pregnancy was tubal and specifically ampullary which is the most frequently observed site.

The intrauterine component of an HP could be a single or multiple gestations, live or dead, could be aborted spontaneously or progress to term safe delivery [5]. In our extensive search of literature using Google scholar, Pubmed, African journal online (AJOL) and HINARI search engines, no case of a blighted ovum as the intrauterine component of an HP had been reported. This case is thus of particularity as it is does not only present an HP in natural conception but also describes a blighted ovum as the intrauterine component of the HP, which is a true rarity.

Tal and colleagues in a review reported that 70% of HPs are diagnosed between 5 and 8 weeks, 20% between 9th and 10th week and remaining 10% from 11th week [2]. The case reported falls in line with the 20% of people diagnosed between 9th and 10th week. An ectopic pregnancy and a blighted ovum have similar presentations of: no symptoms, lower abdominal pain, and vagina bleeding. This was the case with our patient. The similarity in symptoms and rarity of its co-occurrence could make one to preclude the diagnosis of the other.

Diagnosis usually requires a high index of clinical suspicion as patients might be asymptomatic. Confirmation is usually by trans-vaginal or trans-abdominal ultrasonography [12]. A blighted ovum is diagnosed by a trans-vaginal sonographic mean gestational sac diameter (MGSD) of > 20 mm with no foetal pole or MGSD of > 25 mm with no foetal pole on trans-abdominal sonography [13]. The standard would have been a trans-vaginal sonography, this was however not possible due to poor material and human resources. Our patient benefited from a trans-abdominal ultrasound scan which revealed an MGSD of 29.9 mm with no foetal pole confirming a blighted ovum. Similarly, ectopic pregnancy was diagnosed by left adnexal mass on trans-abdominal ultrasonography with a positive pregnancy test.

A blighted ovum could be managed either expectantly or by evacuation. Both methods have been shown to be efficacious with expectant management within 3 weeks being advisable due to decreased risk of infection and complications [14]. However the patient’s choice often takes precedence. In our case, evacuation was done on request of the patient secondary to persistent bleeding. Management of ectopic pregnancies could be medical or surgical. The Fernandez score is used as a guide to decide on mode of management. Medical therapy in HPs with leaving foetus is however controversial, as Methotrexate has undesirable effects on the developing foetus [15]. This patient’s Fernandez score could not be gotten as our facility was unequipped to measure human chorionic gonadotrophin and progesterone levels. Persistent bleeding and unavailability of methotrexate prompted surgical management which was successful.

The case presented confirms that HP can occur in the absence of predisposing factors, and that the detection of a blighted ovum should not preclude the possibility of a simultaneous ectopic pregnancy. We therefore advocate in all pregnant women with first trimester bleeding even in the presence of a confirmed blighted ovum, a complete review of the whole pelvis including adnexa during ultrasound scan.

## Abbreviations

HP: heterotopic pregnancy
MGSD: mid gestation sac diameter
